# Rapid Point-of-Care Tests Using Staphylococcal Protein A Can Detect Early IgM Responses in HIV-1 and Treponema pallidum Infections

**DOI:** 10.1128/spectrum.03309-22

**Published:** 2022-12-01

**Authors:** Valentina A. Schmidt, Victoria Rose Stevens, Javan Esfandiari, Konstantin P. Lyashchenko

**Affiliations:** a Chembio Diagnostic Systems, Inc., Medford, New York, USA; Quest Diagnostics Nichols Institute

**Keywords:** antibody, IgM, protein A, HIV, syphilis, serodiagnosis

## Abstract

Serological assays detecting IgM antibodies in addition to IgG antibodies have a diagnostic advantage in finding early infections. Staphylococcal protein A (SpA), widely used as an antibody-detecting reagent in various immunoassays, is considered to have a high binding affinity mainly to IgG, although its interaction with other classes of immunoglobulins has also been documented. Using 28 samples from 22 HIV-1 seroconversion panels, the present study demonstrated detection of early IgM antibodies by SpA-based rapid point-of-care tests, including DPP HIV 1/2, DPP HIV-Syphilis, STAT-PAK HIV 1/2, and Sure Check HIV 1/2. Samples with predominant IgM antibodies were identified by in-house IgM assays and confirmed by pretreatment with 0.1 M 2-mercaptoethanol. Likewise, the detection of treponemal IgM antibodies was shown by DPP HIV-Syphilis assay in eight samples collected at early syphilis infection. Direct interaction between IgM and SpA immobilized in solid phase or in solution was demonstrated with purified human polyclonal IgM. A strong correlation was found between the antibody levels detected by SpA and anti-IgM reagent in the early seroconversion samples, thus supporting the evidence for IgM binding by SpA. These assays demonstrated the ability to detect IgM antibodies, which may increase test sensitivity in early infections due to a reduced serodiagnostic window.

**IMPORTANCE** Sexually transmitted infections, including HIV and syphilis, remain a global public health concern. The main laboratory testing approach for HIV and syphilis relies on serological assays. Detection of the IgM class of antibodies may have a diagnostic advantage in finding early infections. The present study using well-characterized HIV-1 and syphilis samples has demonstrated that staphylococcal protein A employed for antibody detection in rapid point-of-care tests, including DPP HIV 1/2, DPP HIV-Syphilis, STAT-PAK HIV 1/2, and Sure Check HIV 1/2, can capture IgM antibodies in addition to IgG antibodies. The findings strongly suggest that the ability to detect IgM antibodies by these immunoassays may facilitate the identification of acute-stage HIV and syphilis infections.

## INTRODUCTION

Sexually transmitted infections (STI), including HIV and syphilis, remain a serious public health concern worldwide (1). About 38,000 new HIV-1 infections have been reported in the United States annually between 2014 and 2018 (2). Likewise, the incidence of primary and secondary syphilis is on the rise in the United States for the sixth consecutive year (3), despite the decrease in syphilis testing during the COVID-19 pandemic (4). The World Health Organization estimated 1.5 million new HIV infections in 2020 worldwide, with 37.7 million people living with HIV (1). Globally, approximately 6 million new cases of syphilis are reported annually in the population between 15 and 49 years of age (5). There is also an upsurge in the incidence of HIV/syphilis coinfections, as the two STIs can be viewed as synergistic (6). Thus, 38.5% of reported syphilis cases were coinfected with HIV across all demographic categories in the United States (7).

Serological assays that rely on the antibody responses constitute the main testing approach for HIV and syphilis infections (5). Early diagnosis using rapid point-of-care tests (POCTs) is essential for timely treatment initiation and prevention of new infections. Adaptive IgM antibody is the first pathogen-specific immunoglobulin isotype to emerge during acute illness (8). Therefore, serological assays detecting IgM in addition to IgG antibodies would have a diagnostic advantage in finding early infections.

In HIV-1 infection, the first circulating IgM antibodies to gp41 antigen can be detected as early as 1.5 to 2 weeks following the appearance of viral RNA in plasma and several days before the development of IgG responses (8–10). Therefore, this stage of HIV-1 infection (Fiebig class III) is characterized by a serodiagnostic window when IgM antibody can be found in the absence of IgG antibodies (11), which appear in the blood typically 3 to 4 weeks after the initial positive result obtained by a molecular assay and remain detectable over the course of disease (12).

Serological diagnosis of syphilis relies on combined use of two types of antibody tests in either traditional or reverse algorithms (13). Treponemal immunoassays detect antibodies to protein antigens of Treponema pallidum, a causative agent of syphilis, whereas nontreponemal assays detect antibodies to lipoidal antigens released from host cells damaged by the pathogen. Treponemal assays are generally more sensitive in acute infection when both IgM and IgG antibodies to T. pallidum can be detected around 2 to 4 weeks postexposure (5). Nontreponemal tests, when used in conjunction with treponemal assays, enable the most accurate identification of active syphilis cases, monitoring treatment, and diagnosis of reinfections (13, 14).

Numerous serological tests developed for a variety of infectious diseases have employed staphylococcal protein A (SpA) as a universal reagent for antibody detection (15). Because SpA is well-known to possess a high binding affinity to the Fc fragment of human IgG, SpA-based immunoassays have been traditionally perceived as detecting primarily IgG antibodies (15). Previous studies on the structural and functional characterization of SpA have also revealed its ability to interact with Fab fragments of other immunoglobulin classes, including human IgM representing the V_H_3-encoded family (16, 17).

**FIG 1:**
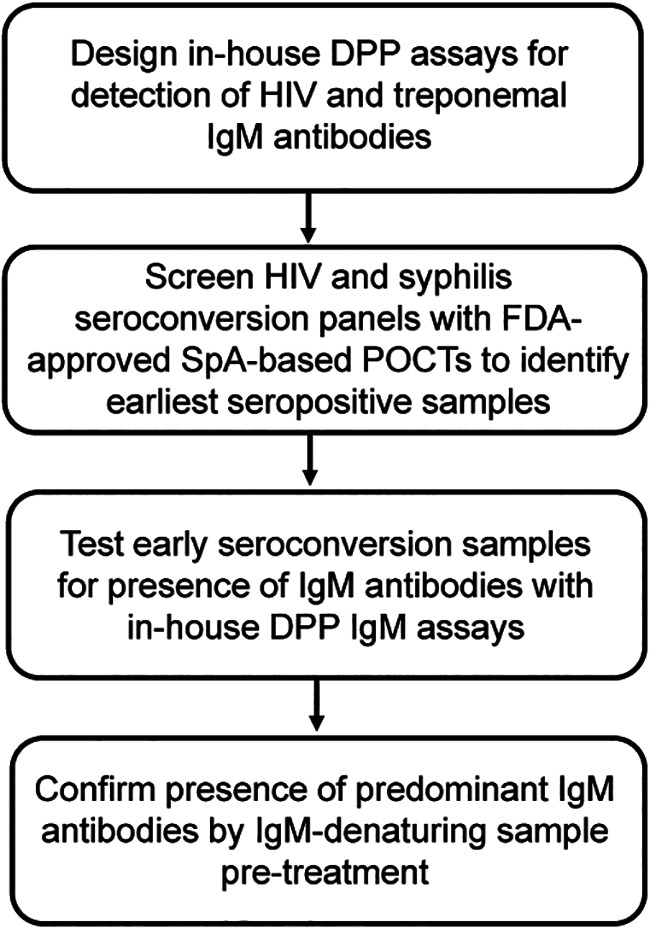
Experimental design used in the study.

Recent reports have demonstrated that commercial HIV POCTs utilizing SpA can detect IgM antibodies in HIV-1 seroconversion panels (18, 19). The present study extends those observations by showing the ability of SpA used in a series of other POCTs for HIV and syphilis to detect IgM antibodies in acute HIV-1 or T. pallidum infections, suggesting a potential for increased test sensitivity in early infection due to a reduced serodiagnostic window. The experimental design is shown in Fig. 1.

## RESULTS

### Direct binding of IgM by SpA in DPP assay format.

**FIG 2:**
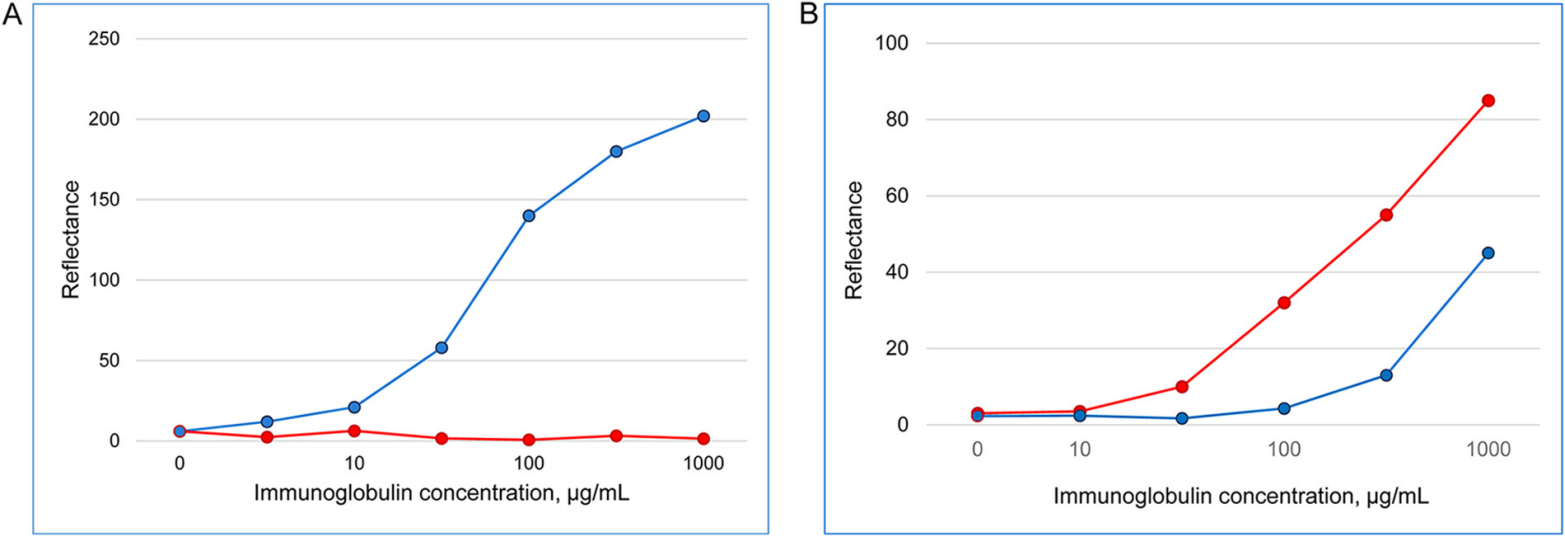
Binding of SpA to human polyclonal IgM or IgG in DPP assays. (A) Purified IgM (blue line) or IgG (red line) evaluated at various concentrations were captured by solid-phase SpA and detected with anti-IgM antibody coupled to gold nanoparticles. (B) Purified IgM (blue line) or IgG (red line) evaluated at various concentrations were captured by solid-phase SpA and detected with SpA coupled with gold nanoparticles. Micro Reader reflectance values are shown in relative light units.

To determine whether SpA used for antibody detection in the DPP HIV 1/2 and DPP HIV-Syphilis assays is capable of binding IgM in addition to IgG, two experimental DPP assays with SpA immobilized onto a membrane strip to serve as a test line for immunoglobulin capture were designed using two different detection reagents. One assay included the anti-IgM gold nanoparticles utilized in the in-house DPP HIV 1/2 IgM test, whereas the other used the SpA-gold nanoparticles employed in the DPP HIV 1/2 assay. Purified human IgM and IgG were tested each at semilogarithmic dilutions to generate dose-response curves. IgM captured by solid-phase SpA produced semiquantitative results with the anti-IgM gold nanoparticles across immunoglobulin concentrations of 10 to 1,000 μg/mL, whereas IgG was undetectable at any concentration in this assay (Fig. 2A), confirming its high isotype specificity. In contrast, both IgM and IgG were detected by the other, “double-SpA sandwich” assay, although approximately one order higher concentrations were required for IgM to yield reading values comparable to those obtained with IgG (Fig. 2B), reflecting different SpA-binding avidities for the two immunoglobulin classes. The results demonstrate the ability of SpA to detect human IgM in the DPP assay format.

### IgM antibody detection in HIV-1 seroconversion panels.

Using the DPP HIV 1/2 and DPP HIV-Syphilis assays, 40 HIV-1 seroconversion panels were tested to determine the earliest seropositive panel members. Once identified, the samples were tested and selected for the presence of predominantly IgM antibodies by the in-house DPP HIV 1/2 IgM assay before and after sample pretreatment with 0.1 M 2-mercaptoethanol (2-ME). Samples producing at least a 50%-reduced Micro Reader signal in both DPP HIV 1/2 and DPP HIV 1/2 IgM assays after the 2-ME treatment were determined as those containing predominantly IgM antibodies.

**FIG 3:**
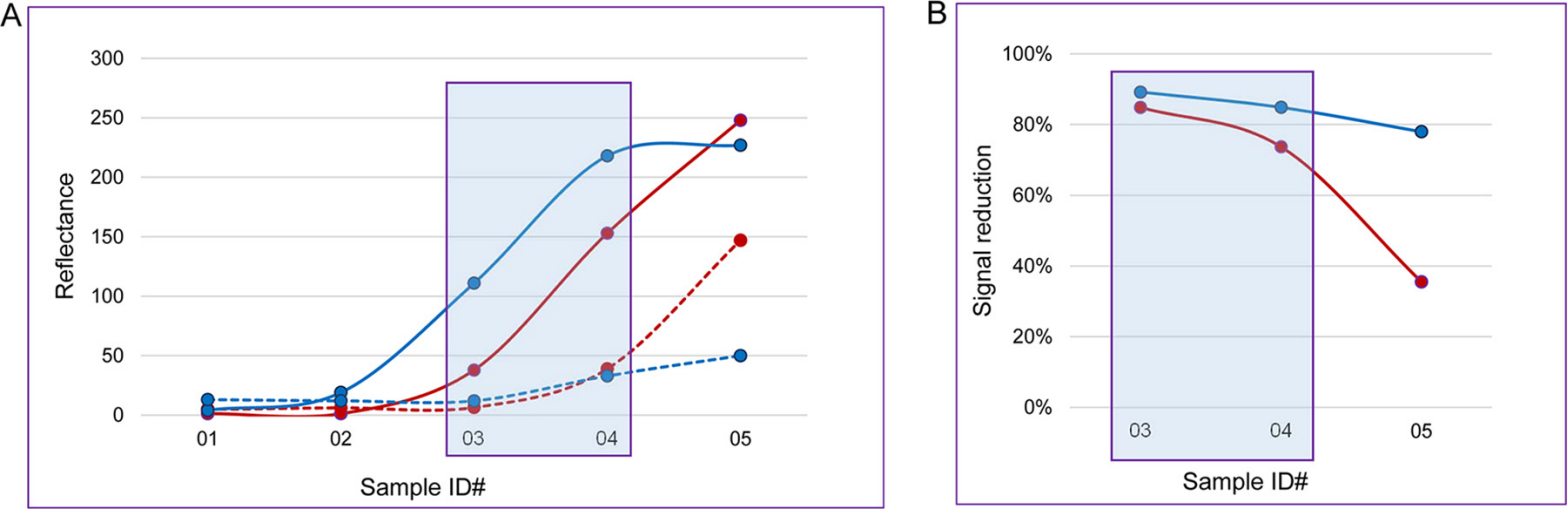
Antibody detection in HIV-1 seroconversion panel PRB-927 and effect of sample pretreatment with 0.1 M 2-ME. (A) Micro Reader reflectance values generated with the DPP HIV 1/2 (red lines) and DPP HIV 1/2 IgM assays (blue lines) are shown for samples tested before (solid lines) and after 2-ME treatment (dashed lines). Framed are test results for samples 03 and 04 collected during initial IgM seroconversion (days 33 and 35 since 1st bleed), in which antibody detection by each of the two immunoassays was diminished after 2-ME treatment by at least 50% signal reduction. (B) Results show differentially evolving susceptibility to the 2-ME treatment of postseroconversion samples tested by the DPP HIV 1/2 (red line) and DPP HIV 1/2 IgM assays (blue line). Framed is the same data subset as that included in panel A but results are shown in percent signal reduction over time.

Figure 3 shows a representative example of HIV-1 seroconversion panel (PRB 927) evaluated using this approach. Samples 03 and 04 met the selection criteria outlined above to support the IgM-binding ability of SpA used in the POCTs. These two samples, collected 2 days apart, constitute an initial seroconversion timespan established by the DPP HIV 1/2 assay, contain IgM antibodies detected by the DPP HIV 1/2 IgM test, and show a significant effect of the 2-ME pretreatment on the result obtained by both immunoassays (Fig. 3). In contrast, SpA-based antibody detection in panel member 05 (collected 5 days after sample 04) was less affected by the IgM-denaturing treatment. A similar trend in the susceptibility to the 2-ME treatment evolving over time (Fig. 3B), suggestive of a growing IgG antibody response, was also observed with other HIV-1 seroconversion panels. Therefore, only early antibody-positive panel members were selected for further analyses.

Following this algorithm (Fig. 1), 22 samples from 19 HIV-1 seroconversion panels were identified as having predominant IgM antibodies. This set was supplemented with 6 IgM-positive samples from 3 HIV-1 seroconversion panels described previously (18), which were not characterized for the presence of IgM antibodies in the present study due to limited sample availability. As a result, the ability of four SpA-based POCTs to detect IgM antibodies was demonstrated with a total of 28 samples from 22 HIV-1 seroconversion panels (Table 1). Depending on the origin and methodology of the IgM data, the samples included in Table 1 were divided into three groups. Groups 1 and 2 included 14 samples previously evaluated for IgM antibodies (18). Groups 2 and 3 consisted of 22 samples for which the predominant IgM antibody status was demonstrated in the present study. Consistent with the DPP HIV 1/2 and DPP HIV-Syphilis data, SURE CHECK HIV 1/2, and STAT-PAK HIV 1/2 tests produced antibody-positive results with all selected samples from groups 1 to 3, except for one sample missed by STAT-PAK HIV 1/2 test (Table 1).

**TABLE 1:** IgM antibody detection by SpA-based rapid point-of-care tests in HIV-1 seroconversion panels^*a*^

Group no.	Evidence for predominant IgM antibody	Sample ID^*a*^	Days since first bleed	Test result			
				DPP HIV 1/2	DPP HIV-Syphilis^*b*^	SURE cHECK HIV 1/2	STAT-PAK HIV ½
Group 1	Described by Moshgabadi et al. (18)	PRB-914-02	4	Positive	Positive	Positive	Positive
		PRB-914-03	7	Positive	Positive	Positive	Positive
		PRB-925-05	44	Positive	Positive	Positive	Positive
		PRB-925-06	49	Positive	Positive	Positive	Positive
		PRB-928-02	111	Positive	Positive	Positive	Positive
		PRB-940-04	15	Positive	ND^*c*^	Positive	Positive
Group 2	Described by Moshgabadi et al. (18); confirmed in present study with DPP HIV 1/2 IgM assay	PRB-914-01	0	Positive	Positive	Positive	Positive
		PRB-924-07	35	Positive	Positive	Positive	Negative
		PRB-924-08	40	Positive	Positive	Positive	Positive
		PRB-927-03	33	Positive	Positive	Positive	Positive
		PRB 927-04	35	Positive	Positive	Positive	Positive
		PRB-934-02	7	Positive	Positive	Positive	Positive
		PRB-934-03	11	Positive	Positive	Positive	Positive
		PRB-944-05	14	Positive	Positive	Positive	Positive
Group 3	Demonstrated in present study with DPP HIV 1/2 IgM assay	PRB-909-03	14	Positive	Positive	Positive	Positive
		PRB-912-02	9	Positive	Positive	Positive	Positive
		PRB-917-05	65	Positive	Positive	Positive	Positive
		PRB-929-07	28	Positive	Positive	Positive	Positive
		PRB-930-04	10	Positive	Positive	Positive	Positive
		PRB-933-03	27	Positive	Positive	Positive	Positive
		PRB 945-06	20	Positive	Positive	Positive	Positive
		PRB-967-06	24	Positive	Positive	Positive	Positive
		9014-03	10	Positive	Positive	Positive	Positive
		9018-11	36	Positive	Positive	Positive	Positive
		9075-04	33	Positive	Positive	Positive	Positive
		12007-08	132	Positive	Positive	Positive	Positive
		12008-12	41	Positive	Positive	Positive	Positive
		SCP-HIV-004-05	56	Positive	Positive	Positive	Positive

a Included are IgM-positive samples that are susceptible to 2-ME pretreatment causing ≥50% DPP signal reduction.

b Results are shown for HIV antibodies only.

c ND, not done.

### IgM antibody detection in syphilis plasma samples.

**FIG 4:**
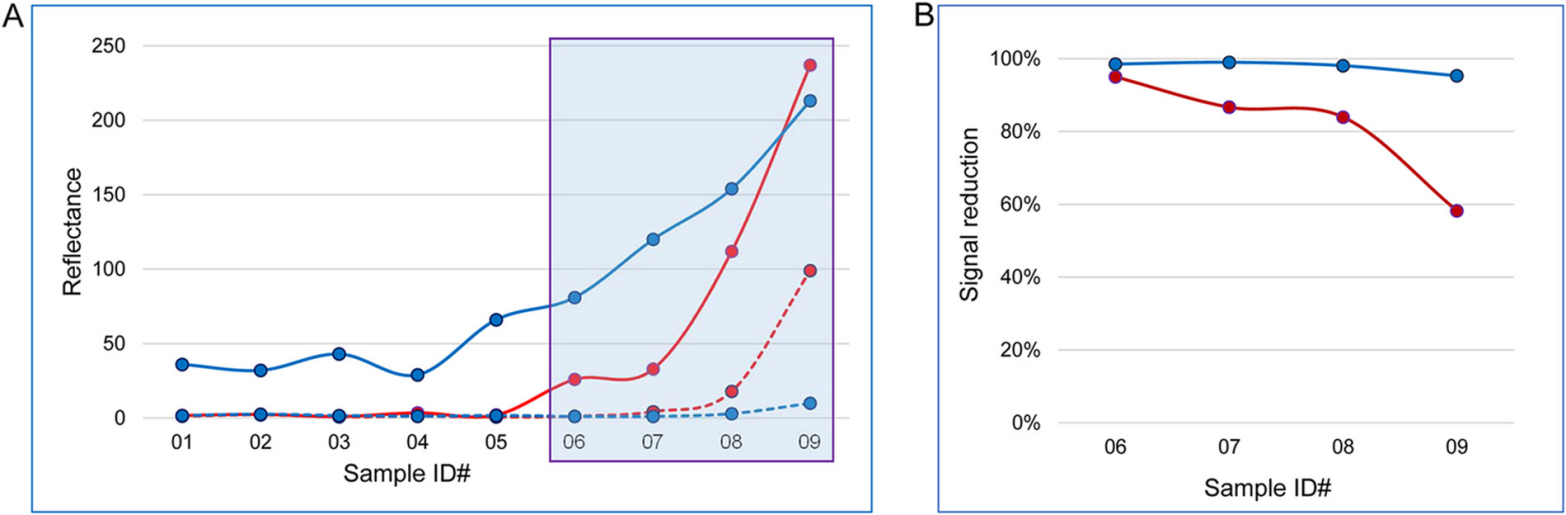
Antibody detection in AccuVert Syphilis Seroconversion Panel PSS901 and effect of sample pre-treatment with 0.1 M 2-ME. (A) Micro Reader reflectance values generated with the DPP HIV-Syphilis (red lines) and DPP HIV-Syphilis IgM assays (blue lines) are shown for samples tested before (solid lines) and after 2-ME treatment (dashed lines). Framed are test results for samples 06 to 09 collected during the initial IgM response (days 45 to 59 since 1st bleed), in which antibody detection with each of the two immunoassays was diminished after 2-ME treatment by at least 50% signal reduction. (B) Results show differentially evolving susceptibility to the 2-ME treatment of postseroconversion samples (same as framed in panel A) tested by the DPP HIV-Syphilis (red line) and DPP HIV-Syphilis IgM assays (blue line).

A similar approach described for the HIV-1 sample selection was applied to identify samples representing active syphilis infection characterized by predominant IgM antibodies to T. pallidum. Evaluation of AccuVert PSS901 syphilis seroconversion panel, which consisted of nine members, by the DPP HIV-Syphilis and in-house DPP HIV-Syphilis IgM assays yielded antibody-positive results with samples 06 through 09 collected over a span of 2 weeks, indicating a growing humoral immune response (Fig. 4A). Sample pretreatment with 2-ME led to significantly diminished reflectance values in both assays (Fig. 4B), displaying a similar trend over time to that observed with the HIV-1 seroconversions (Fig. 3B).

Additional candidates for treponemal IgM antibody characterization were identified as follows. When initially screening HIV-1 seroconversion panels, antibodies to both HIV-1 and T. pallidum were found by the DPP HIV-Syphilis assay in two panels, PRB-912 and PRB-933, suggestive of possible coinfections. Treponemal IgM antibodies were detected by the DPP HIV-Syphilis IgM assay in panel PRB 933 but not in PRB-912. Further, testing of 26 plasma samples collected at unknown stages of active syphilis (all rapid plasma reagin [RPR] positive) yielded three samples with treponemal IgM antibodies detected by the DPP HIV-Syphilis IgM assay. As a result, a total of eight syphilis samples showing predominant IgM antibodies that are highly susceptible to 2-ME treatment were identified (Table 2). For comparison, two representative examples of DPP HIV-Syphilis antibody-positive but IgM-negative samples were included to demonstrate the resistance of IgG antibodies to the IgM-denaturing method used in the present study (Table 2).

**TABLE 2:** IgM antibody detection by DPP HIV-Syphilis assay in active syphilis^*a*^

Sample ID	Days since first bleed	RPR		Testing prior to 2-ME treatment				DPP signal reduction after 2-ME treatment	
				DPP HIV-Syphilis		DPP HIV-Syphilis IgM		DPP HIV-Syphilis	DPP HIV-Syphilis IgM
		Result	Titer	Result^*b*^	s/co^*c*^	Result^*b*^	s/co^*c*^		
PSS901-06	45	Positive	1:8	Positive	3.7	Positive	1.4	95%	99%
PSS901-07	48	Positive	1:16	Positive	4.7	Positive	2.0	87%	99%
PSS901-08	52	Positive	1:32	Positive	16.0	Positive	2.6	84%	98%
PSS901-09	59	Positive	1:64	Positive	33.9	Positive	3.6	58%	95%
w36981501276400	NA	Positive	1:1	Positive	5.4	Positive	1.4	91%	100%
w36981501452200	NA	Positive	1:16	Positive	30.7	Positive	2.6	68%	97%
w36981502181900	NA	Positive	1:1	Positive	30.9	Positive	1.4	62%	99%
PRB-933-02	21	ND	NA	Positive	1.3	Positive	1.2	87%	98%
DLS0116358	NA	Positive	1:64	Positive	36.6	Negative	0.9	16%	NA
PRB-912-02	9	ND	NA	Positive	5.7	Negative	0.3	8%	NA

a RPR, rapid plasma reagin; s/co, signal-to-cutoff ratio; ND, not done; NA, not applicable.

b Results are shown for treponemal antibodies, all samples were HIV antibody negative, except for PRB-933-02 and PRB-912-02.

c Test interpretation: positive result at signal-to-cutoff ration (s/co) ≥1.0, negative result at s/co <1.0.

Pretreatment of syphilis samples with 2-ME abolished treponemal IgM signal by 95 to 100% (Table 2). Antibody levels detected by SpA used in the DPP HIV-Syphilis assay were diminished upon 2-ME treatment as well, albeit to a lower extent, with signal reduction ranging from 58% to 95%. The degree of signal reduction appeared to be associated with the RPR titers (*r* = −0.71), thus inferring that the selected samples with high treponemal IgM levels represent early stages of active T. pallidum infections. In contrast, IgM-negative syphilis samples showed negligible signal reduction upon 2-ME treatment (Table 2).

### Correlation between SpA-detected antibodies and IgM antibodies in early HIV-1 and T. pallidum infections.

**FIG 5:**
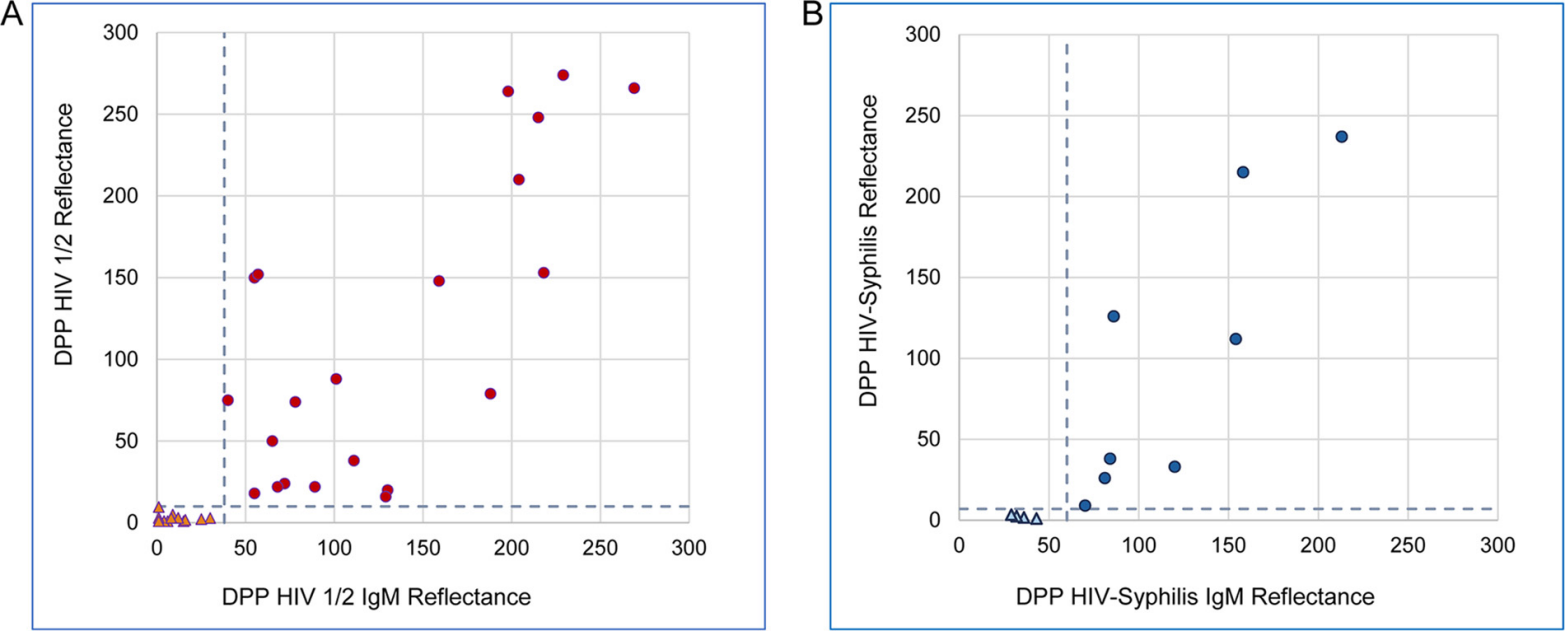
Correlation between antibody levels detected by SpA or anti-IgM reagent in early HIV-1 seroconversion and syphilis samples. (A) Micro Reader reflectance values generated with the DPP HIV-1/2 and DPP HIV-1/2 IgM assays are shown for the HIV-1 seroconversion samples included in Table 1, Groups 2 and 3 (circles), and matching preseroconversion members (triangles). (B) Micro Reader reflectance values generated for treponemal antibody levels with the DPP HIV-Syphilis and DPP HIV-Syphilis IgM assays are shown for the syphilis-positive samples listed in Table 2 (circles) and preseroconversion panel members (triangles). Horizontal and vertical dashed lines indicate cutoff values used for test interpretation: DPP HIV-1/2, 10 RLU; DPP HIV-1/2 IgM, 40 RLU; DPP HIV-Syphilis, 7 RLU; DPP HIV-Syphilis IgM, 60 RLU.

DPP Micro Reader values generated from the 22 selected HIV-1 samples (Table 1, groups 2 and 3) tested by both DPP HIV 1/2 and DPP HIV 1/2 IgM assays were used to evaluate a relationship between the two test results. A strong positive correlation (*r* = 0.83) was revealed (Fig. 5A), suggesting that the two immunoassays can detect essentially the same pool of antibodies, provided IgM is the predominant isotype in the sample. Preseroconversion samples from the same panels tested antibody negative by both SpA-based DPP HIV1/2 and DPP HIV1/2 IgM assays (Fig. 5A). Likewise, a strong correlation (*r* = 0.89) was found between treponemal antibody levels detected by the DPP HIV-Syphilis and DPP HIV-Syphilis IgM assays in the 8 selected samples (Fig. 5B), whereas the preseroconversion samples tested antibody-negative by both immunoassays.

## DISCUSSION

The present study evaluated the ability of SpA used in rapid POCTs to detect IgM responses in early HIV-1 and T. pallidum infections. A direct interaction between SpA and IgM in the DPP assay format was demonstrated in a proof-of-principle experiment using purified human polyclonal IgM, with a dynamic range of semiquantitative IgM detection being comparable to the reference range for normal IgM levels in human blood (20). Early seroconversion samples collected in acute HIV-1 infection (*n* = 28) or syphilis (*n* = 8) with predominant IgM antibodies were identified using in-house DPP IgM assays and confirmed by 2-ME treatment. Testing these samples by DPP HIV 1/2, DPP HIV-Syphilis, STAT-PAK HIV 1/2, and Sure Check HIV 1/2, each employing SpA-gold nanoparticles, produced antibody-positive results in all four POCTs, demonstrating their ability to detect IgM responses developed prior to IgG-mediated seroconversion. The close correlation observed between antibody levels detected by SpA and anti-IgM reagent in the early seroconversion samples further supported the evidence for IgM binding by SpA.

These findings are in agreement with earlier reports demonstrating that other SpA-based POCTs, the INSTI HIV-1/HIV-2 Antibody Test (18) and the OraQuick ADVANCE Rapid HIV 1/2 Antibody Test (19), could detect IgM antibodies in select samples from 12 HIV-1 seroconversion panels. The present study extends the previously reported observations in the following areas: (i) direct binding of human IgM to SpA on solid phase and in solution was analyzed in comparison with IgG by the two experimental DPP assays of different configurations; (ii) alternative approach for selective inactivation of IgM antibodies (2-ME treatment) was applied to confirm the isotype specificity; (iii) a large sample size (28 specimens from 22 HIV-1 seroconversion panels), half of which had never been tested before for the presence of predominant IgM antibodies, was collected and characterized; (iv) four distinct rapid POCTs using SpA in various immunoassay configurations were demonstrated to detect early IgM antibodies; (v) in addition to the HIV-1 antibody assays, the ability of SpA-based DPP HIV-Syphilis assay to detect IgM antibodies to T. pallidum was shown with plasma samples collected during active syphilis infection; and (vi) an optical reader instrument was used for semiquantitative evaluation of antibody reactivity in HIV-1 and syphilis samples, thus enabling accurate measurements for objective test interpretation, sample identification and grouping based on defined selection criteria, and statistical data analyses.

To confirm the isotype specificity of IgM detection by SpA, samples were pretreated with 0.1 M 2-ME, a reducing agent known to disrupt disulfide bonds connecting monomers in IgM molecules, thus abolishing their antigen-binding ability (21–23). This proven method has been extensively used in serological analyses to selectively denature IgM in a variety of mammalian species, including humans, mice, and cattle (21, 24, 25) without affecting the integrity and functionality of other immunoglobulin classes, including IgG (22, 23). Therefore, a meaningful decrease of detectable antibody levels after 2-ME treatment (≥50% signal reduction adopted in the present study) can be attributed to a high IgM prevalence in the antibody pool detected by SpA. The present study results produced using this approach are concordant with the previously reported data obtained with some of the same HIV-1 seroconversion samples tested before and after IgM removal by immunodepletion (18), thereby bridging the two methods for IgM elimination.

SpA produced by Staphylococcus aureus in both secreted and cell wall-associated forms is a potent virulence factor capable of targeting the immune system as a superantigen that induces exhaustive polyclonal activation of B cells (15, 17, 26). Contributing to the pathogenesis of infection, this effect of SpA is mediated by its natural ability to closely interact with cell-surface and circulating immunoglobulins, primarily of IgG class but also other immunoglobulin classes, in humans and animals (27, 28). The distinct feature of having unconventional binding affinity to mammalian immunoglobulins has uniquely positioned SpA for various applications in immunobiological research and biomedical technologies, including immunodiagnostics and affinity chromatography (15, 29).

SpA is a 42-kDa protein consisting of five domains (E, D, A, B, and C) that are involved in binding the C2-C3 domain of the Fc region in human IgG1, IgG2, and IgG4, as well as the Fab region of IgM, IgG, IgA, and IgE (17, 28). Importantly, the Fab-binding sites on immunoglobulins are distant from complementarity-determining regions, facilitating noncompetitive antigen-antibody interactions when occurring in the presence of SpA (15). The ability to bind SpA is restricted to a subgroup of human IgM molecules containing a V_H_3 H chain to the extent that binding to SpA can be used as a functional marker distinguishing V_H_3 IgM from V_H_1 and V_H_2 IgM (27, 30). While the V_H_3-encoded IgM subgroup represents the largest human V_H_ gene family, the SpA binding to IgM is generally variable due to the immunogenetic heterogeneity of the human population (16, 17). Combined with the restriction of SpA binding to the V_H_3 subgroup, this may explain why low-level IgM antibodies developed in early infections are not always detectable by SpA-based antibody tests.

A timely diagnosis is essential for the most efficient treatment and disease control. Rapid POCTs offer several advantages for improving disease control compared to the standard clinical laboratory-based tests, such as a shorter time to result (under 30 min), extended accessibility, ease of use, and cost-effectiveness (31). IgM is known to be the earliest antibody class to emerge in the adaptive immune responses to acute infections (8). Therefore, antibody tests capable of IgM detection may facilitate early serodiagnosis.

The four rapid POCTs evaluated in this study for the ability to detect IgM antibodies are all FDA-approved products. SURE CHECK HIV 1/2 test is also approved in Europe and Brazil as an over-the-counter product for home self-testing. A recent evaluation study, conducted by the U.S. Centers of Disease Control and Prevention using a large collection of well-characterized HIV-1 and HIV-2 samples collected at various stages of acute infections, has demonstrated that the DPP HIV 1/2 assay can detect antibodies 4 to 5 days earlier than several other commercial POCTs (9), consistent with the findings presented here.

In conclusion, the present study demonstrated the ability of four SpA-based POCTs to detect early IgM responses in HIV-1 infection as well as in active syphilis. The results imply that the serodiagnostic window, generally observed with immunoassays relying on the detection of IgG antibodies only, may be reduced when using tests employing SpA. The reported findings may have important implications for serological testing of other infectious diseases, for which detection of IgM is beneficial for earlier diagnosis.

## MATERIALS AND METHODS

### Samples.

A total of 40 HIV-1 seroconversion panels (SeraCare Diagnostics, West Bridgewater, MA, USA; Zeptometrix, Inc., Buffalo, NY, USA; Biomex GmbH, Heidelberg, Germany) were evaluated with the immunoassays described below following the experimental design shown on Fig. 1. Syphilis seroconversion panel AccuVert PSS901 consisting of 9 members (SeraCare Diagnostics, West Bridgewater, MA, USA) was tested along with additional 26 plasma samples from patients diagnosed with active syphilis (MRN Diagnostics, Franklin, MA, USA; Discovery Life Sciences, Inc., Huntsville, AL, USA).

### Rapid plasma reagin test.

RPR titers were determined using ASI RPR card test for syphilis (Arlington Scientific, Inc., Springville, UT, USA) and the manufacturer’s instructions.

### Rapid point-of-care tests.

The following FDA-approved rapid POCTs manufactured by Chembio Diagnostic Systems, Inc. (Medford, NY, USA) were included in the study: (i) STAT-PAK HIV 1/2 Assay; (ii) Sure Check HIV 1/2 Assay; (iii) DPP HIV 1/2 Assay; and (iv) DPP HIV-Syphilis Assay. The first two are conventional lateral-flow assays, whereas the other two were developed using the Dual-Path Platform (DPP) technology. All four assays use SpA-coupled colloidal gold nanoparticles for antibody detection. Testing was conducted according to the manufacturer’s instructions for each product. Results for all assays were recorded as antibody positive or antibody negative based on visual interpretation. To generate semiquantitative data in the DPP products, an optical instrument Micro Reader was used to measure test line reflectance values expressed in relative light units (RLU).

### In-house DPP assays for IgM antibodies.

DPP HIV 1/2 and DPP HIV-Syphilis tests were converted into investigational IgM-detecting assays, named DPP HIV 1/2 IgM and DPP HIV-Syphilis IgM, respectively, by replacing SpA coupled to gold nanoparticles with mouse monoclonal antibody to human IgM (HyTest Ltd., Turku, Finland), a specific reagent for IgM detection validated in commercial DPP products (14, 32). Plasma samples were tested for the presence of IgM antibodies following the assay procedure recommended by the manufacturer for the respective SpA-based DPP products. Semiquantitative results were generated using DPP Micro Reader to measure test line reflectance. Test interpretation was based on the preestablished cutoff values of 40 RLU for DPP HIV 1/2 IgM and 60 RLU for DPP HIV-Syphilis IgM assays.

### Experimental DPP assays for total IgM and IgG.

Nitrocellulose membrane printed with SpA was used as a test strip in two experimentally designed DPP assays, with each using one of the two distinct reagents, SpA and anti-IgM antibody, coupled to gold nanoparticles as described above. These two DPP assay versions were used to characterize the direct binding of IgM by SpA immobilized on the solid phase by testing purified human polyclonal IgM and IgG (Sigma, St. Louis, MO, USA) at serial dilutions following the assay procedure recommended by the manufacturer for the other DPP tests described above. Semiquantitative results were generated with the DPP Micro Reader.

### IgM inactivation.

Plasma samples were mixed at 1:1 ratio with 0.2 M 2-ME (Sigma, St. Louis, MO, USA) prepared in phosphate-buffered saline, resulting in 0.1 M final concentration of 2-ME, and incubated at 37°C for 1 h (21, 24). Following the 2-ME treatment, samples were immediately tested in parallel with corresponding untreated samples by the immunoassay described above.

### Data analysis.

The cutoff value for the in-house IgM DPP assays was established in a pilot study with 20 known HIV-negative and syphilis-negative samples as the mean plus two standard deviations using DPP Micro Reader-generated data. In IgM inactivation experiments, samples that showed a ≥50% signal decrease in both the SpA-based and IgM detection assays were considered to be IgM antibody predominant. Cutoff calculations and correlation analyses were performed using MS Excel.
